# Air Leak Syndrome: Pneumoperitoneum in a Ventilated Neonate

**DOI:** 10.1155/2019/4238601

**Published:** 2019-12-27

**Authors:** Chandana Ravikumar, Dawn McDaniel, Amy Quinn

**Affiliations:** University of Texas Health San Antonio, San Antonio, TX, USA

## Abstract

Air leak syndrome has several manifestations and is common in neonates with meconium aspiration syndrome (MAS) due to air trapping. While pneumoperitoneum is classically a result of intestinal perforation, intra-abdominal free air may be a less common presentation of air leak syndrome. In the ventilated neonate, there is insufficient clinical evidence outlining management of pneumoperitoneum in this situation. We report a case of a term neonate with MAS and air leak syndrome who developed benign pneumoperitoneum (BPPT).

## 1. Introduction

Air leak syndrome is the overarching diagnosis for one or more pathologic air collections present outside of the respiratory tract. Seven classic presentations in neonates include pulmonary interstitial emphysema (PIE), pneumothorax, pneumomediastinum, pneumopericardium, pneumoperitoneum, subcutaneous emphysema, and systemic air embolism. Air leak occurs in approximately 10% of neonates with MAS [1] for many presumed reasons including ball-valve effect of the meconium, ventilation-perfusion mismatch, surfactant inactivation, and parenchymal injury. While pneumoperitoneum is pathognomonic of perforation of an abdominal viscus [2], it rarely can result from air leak (incidence unknown) and, in such cases, is referred to as benign pneumoperitoneum (BPPT). Management of pneumoperitoneum resulting from a perforated viscus versus BPPT is quite different, the former representing a surgical emergency and the latter allowing the potential for conservative, nonoperative management. In a ventilated neonate with pneumoperitoneum, existing clinical evidence is ambiguous at delineating which cases may be amenable to initial observation alone. For clinicians caring for ill neonates, increased awareness of BPPT as a clinical entity might circumvent unnecessary surgeries and their inherent complications. We report a case of a term neonate with meconium aspiration syndrome (MAS) and air leak syndrome who developed BPPT.

## 2. Case Presentation

An appropriate for gestational age (AGA) female infant was born at 39 6/7 weeks gestation by cesarean section to a 22-year-old Vietnamese mother. The infant was a result of her second pregnancy, the first being a spontaneous abortion. Spontaneous rupture of membranes occurred 26 minutes prior to delivery with thick meconium-stained amniotic fluid. Urgent cesarean section was performed for nonreassuring fetal heart tones. At birth, the infant appeared pale and was covered with thick meconium. She had shallow, regular breaths, a scaphoid abdomen, and otherwise normal tone. After suctioning and stimulation, continuous positive airway pressure (CPAP) and oxygen were inadequate in improving respiratory status as evidenced by worsening bradycardia. Intubation and endotracheal positive pressure ventilation (PPV) at 8 minutes of life resulted in improved color and heart rate.

On admission to the neonatal intensive care unit (NICU), the infant was briefly placed on synchronized intermittent mandatory ventilation (SIMV) but quickly transitioned to high-frequency oscillatory ventilation (HFOV) due to continued hypercapnea and concern for MAS. Initial settings were of amplitude (AMP) 30, frequency (Hz) 10, mean airway pressure (MAP) 18, inspiratory time (iT) 33%, and 60% FiO_2_. Inhaled nitric oxide (iNO) at 20 ppm was initiated. Initial chest x-ray (CXR) demonstrated diffuse bilateral streaky airspace opacities consistent with MAS. Pre-and post-ductal saturations revealed no significant splitting on iNO. At 6 hours of life, a dopamine drip and stress dose hydrocortisone were administered to treat hypotension. Severe coagulopathy necessitated several transfusions of fresh frozen plasma, platelets, and packed red blood cells.

**Figure 1:**
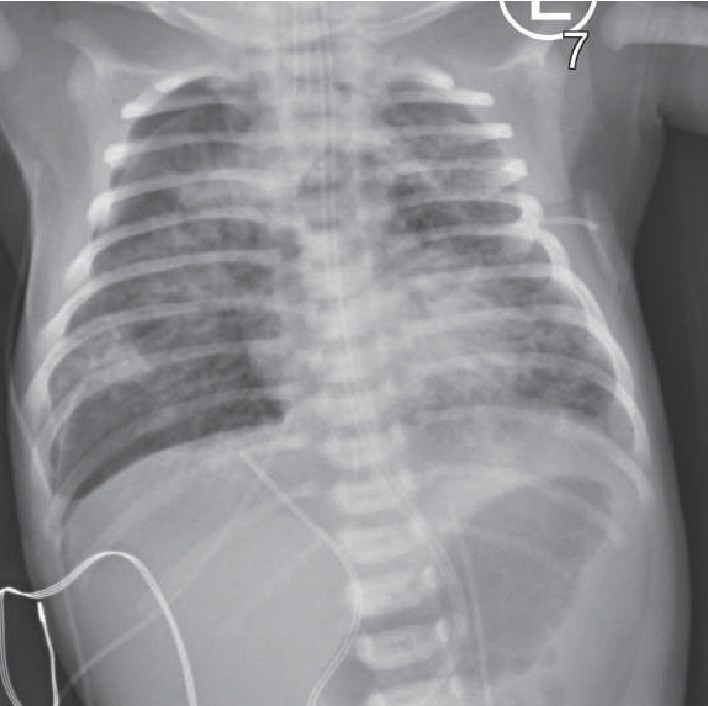
Chest radiograph revealing moderately sized right-sided pneumothorax and pneumomediastinum. Diffuse coarse bilateral pulmonary opacities consistent with MAS.

At 8 hours of life, a CXR revealed a moderately sized right-sided pneumothorax and pneumomediastinum consistent with air leak syndrome as depicted in Figure 1. Ventilator settings were gradually increased due to persistent respiratory acidosis, hypercapnea, and hypoxemia to maximal HFOV settings at 10 hours of life of AMP 43, Hz 6, and MAP 21 with 100% FiO_2_. Echocardiogram showed a patent foramen ovale (PFO) and patent ductus arteriosus (PDA) with low velocity left to right shunting and without notable tricuspid regurgitation jet.

**Figure 2:**
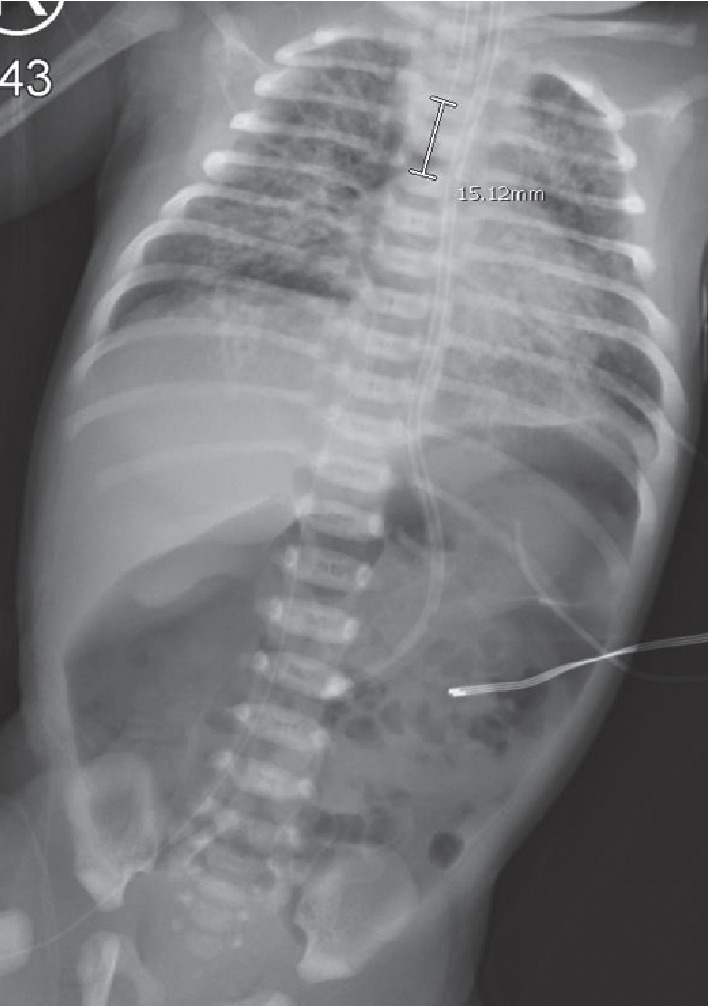
Babygram depicting interval development of a large pneumoperitoneum and placement of right lower extremity PICC.

**Figure 3:**
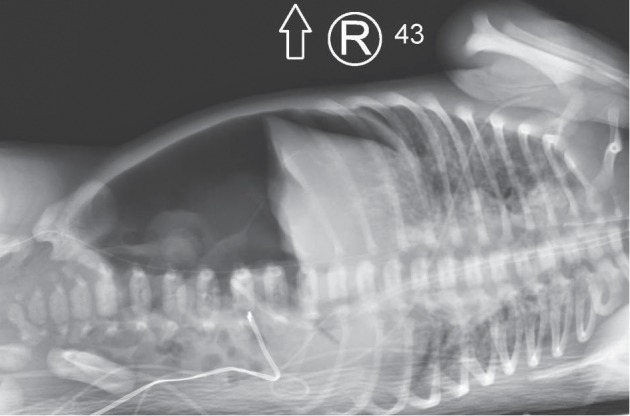
Lateral decubitus radiograph depicting a large pneumoperitoneum.

At 16 hours of life, a babygram obtained for peripherally inserted central catheter (PICC) placement showed an abnormal air collection in the abdomen as shown in Figure 2. A lateral decubitus x-ray clearly demonstrated pneumoperitoneum as seen in Figure 3. HFOV settings were AMP 38, Hz 6, MAP 20, iT 33%, and 85% FiO_2_. On physical examination, the abdomen was soft with mild distention and no discoloration. The pediatric surgical team was consulted and performed an urgent bedside exploratory laparotomy to evaluate for gastric perforation given large size of pneumoperitoneum. During surgery, bubbles were seen emanating from behind the liver suggesting air tracking from the chest into the abdominal cavity. The bowel was “run,” and no visceral perforation was found. A 1/4 inch Penrose drain was placed in the left upper quadrant with minimal serosanguinous fluid drained. Empiric ampicillin and gentamicin was switched to piperacillin-tazobactam for 5 days of postoperative therapy.

The patient's pneumoperitoneum was ultimately determined to be secondary to barotrauma. Postoperative imaging showed resolution of the pneumoperitoneum. Patient was NPO with a Replogle tube in place.

On the third day of life, subcutaneous crepitus was palpated on upper chest and neck. A babygram demonstrated bilateral subcutaneous emphysema in cervical soft tissue. Ventilator settings were AMP 38, Hz 8, and MAP 18.5. The MAP was decreased stepwise over several hours to 15.5 in attempt to alleviate subcutaneous air trapping. Echocardiogram showed a PFO with left to right shunting, mildly dilated right ventricle, and near suprasystemic pulmonary artery pressures for which Milrinone was started.

By the fifth day of life, there was resolution of the pneumothorax, pneumomediastinum, and subcutaneous emphysema. The patient was extubated to high-flow nasal cannula on day of life fourteen. She was treated for opiate withdrawal for several days after discontinuation of sedation medications. Oral feeds were initiated on day of life ten. The Penrose drain was removed on day of life thirteen. She was transitioned to room air on day of life nineteen and discharged from the hospital on her twenty-seventh day of life.

Of note, this patient was readmitted to the pediatric inpatient floor at 2.5 months of age for management of small bowel obstruction, necrotic bowel, and intraperitoneal abscesses secondary to extensive adhesions likely related to her previous exploratory laparotomy. 8 centimeters of jejunum was resected with primary reanastomosis during the surgery. She tolerated slow advancement from TPN to full feeds and was discharged after a seventeen-day hospital stay.

## 3. Discussion

A cascade of events contributed to our patient's clinical course. Severe respiratory failure resulting from MAS necessitated high oscillatory ventilator settings to normalize the pH and achieve adequate oxygenation and ventilation. Combined, the underlying lung injury exacerbated by the ventilator settings led to multiple presentations of air leak including pneumothorax, pneumomediastinum, pneumoperitoneum, and subcutaneous emphysema. Pneumoperitoneum in neonates is most commonly a surgical emergency, the result of visceral perforation in 90% of cases [3]. However, BPPT should remain on the differential diagnosis of the clinician in the appropriate patient population.

Air leak is common in neonates with MAS and who sustain barotrauma/volutrauma from mechanical ventilation. Pneumothorax and PIE are the most common presentations of air leak syndrome in the ventilated neonate [4]. Air leak is caused by overinflation of the alveolus that eventually ruptures. Air subsequently accumulates outside the pleural space in the interstitium. This free air can escape the thorax and cause various presentations of air leak syndrome. BPPT is postulated to be a result of air tracking from a pneumothorax via diaphragmatic foraminae and accumulating in the peritoneal cavity.

BPPT, also referred to as spontaneous idiopathic pneumoperitoneum and nonsurgical pneumoperitoneum, is a rare presentation of air leak syndrome. BPPT is typically seen in conjunction with other classic presentations of air leak and much less likely as an isolated finding. A paucity of data exist to direct management in cases where BPPT is highly suspected. In 1966, a historical study concluded that every neonate with pneumoperitoneum must undergo an exlap [5]. However, over the past few decades clinicians have seen success with conservative medical management of BPPT. Undoubtedly, pneumoperitoneum from a ruptured viscus has grave implications and requires immediate surgical intervention. Nonetheless, given the inherent risks of surgery, the clinician should consider BPPT in patients with evidence of air leak syndrome and who are otherwise at low risk for visceral perforation.

Diagnosis of BPPT is based on history, physical exam, laboratory values, and abdominal radiographs. In addition, abdominocentesis may be considered for diagnostic aid. Clinical manifestations of BPPT include a soft abdomen without distension. There should be no signs of peritoneal irritation or abdominal wall color change. Tension pneumoperitoneum should not be present [6]. Decreasing platelet counts and leukocytosis are associated with perforated viscus and should guide clinicians toward surgical management [7].

One retrospective case review evaluated nine children with BPPT (ages four days to four years). Of these nine cases, eight were managed conservatively with success [3]. Physical examination revealed all patients with soft abdomen except one patient with abdominal distension, slight abdominal muscle stiffness, and hyperactive bowel sounds. Factors used to determine necessity of surgical exploration were age, fever, abdominal signs, leukocytosis, and C-reactive protein. One patient with high index of suspicion for viscus perforation had an exploratory laparotomy that revealed no perforation and was diagnosed with BPPT postoperatively. All nine patients were discharged home. Follow-up assessments after discharge at 7 months to 6 years were conducted with all patients showing good growth without complications.

If the index of suspicion for BPPT remains high after a thorough clinical evaluation, nonsurgical management should be considered, including conservative management, pleurocentesis, abdominocentesis, and optimization of ventilator settings. Conservative management without surgical intervention entails frequent abdominal exams with measurement of abdominal girth and assessment for signs of peritonitis and/or tension pneumoperitoneum which would indicate the need for surgical intervention. In cases where a pneumothorax is concurrently present, pleurocentesis may be performed. Serial radiographs should be performed. Abdominocentesis can be diagnostic and therapeutic. Abdominocentesis resulting in air without fluid would be consistent with BPPT. Abdominocentesis can relieve marked abdominal distension and improve respiratory distress [8]. In ventilated patients, optimization of ventilatory management can alleviate air trapping.

## 4. Conclusion

Intestinal perforation is the most common cause of pneumoperitoneum in the neonate and must be promptly identified and treated. Air leak syndrome is a common complication in ventilated neonates with resultant increase in morbidity and mortality. BPPT, though an uncommon cause of pneumoperitoneum, is a rare presentation of air leak syndrome. In ventilated neonates with pneumoperitoneum and evidence of other more common presentations of air leak syndrome, BPPT should be suspected. Diagnosis of BPPT relies heavily on clinical symptoms, abdominal exam, and radiography. BPPT can be managed medically. Increased awareness of BPPT as a clinical entity may result in fewer unnecessary surgical interventions in neonates with pneumoperitoneum.
